# A Pilot Study for the Detection of Cyclic Prolyl-Hydroxyproline (Pro-Hyp) in Human Blood after Ingestion of Collagen Hydrolysate

**DOI:** 10.3390/nu10101356

**Published:** 2018-09-22

**Authors:** Yasutaka Shigemura, Yu Iwasaki, Mana Tateno, Asahi Suzuki, Mihoko Kurokawa, Yoshio Sato, Kenji Sato

**Affiliations:** 1 Department of Nutrition, Faculty of Domestic Science, Tokyo Kasei University, 1-18-1 Kaga, Itabashi-ku, Tokyo 173-8602, Japan; k151907@tokyo-kasei.ac.jp (Y.I.); tateno-m@tokyo-kasei.ac.jp (M.T.); satouy@tokyo-kasei.ac.jp (Y.S.); 2 Q’sai Co., Ltd., 1-7-16 Kusagae, Chuo-ku Fukuoka City 810-8606, Japan; a_suzuki@kyusai.co.jp (A.S.); kurokawa@kyusai.co.jp (M.K.); 3 Division of Applied Biosciences, Graduate School of Agriculture, Kyoto University, Kitashirakawa Oiwake-Cho, Kyoto 606-8502, Japan; kensato@kais.kyoto-u.ac.jp

**Keywords:** cyclic Pro-Hy, Pro-Hyp, collagen hydrolysate, collagen peptides, fibroblasts, mouse skin

## Abstract

Levels of short linear hydroxyproline (Hyp)-containing peptides, such as prolyl-hydroxyproline (Pro-Hyp), increase in human blood after the ingestion of collagen hydrolysate, which has been associated with beneficial effects for human skin and joints. The present study demonstrates the presence of a novel food-derived collagen peptide, cyclic Pro-Hyp, in human blood after the ingestion of collagen hydrolysate. The cyclic Pro-Hyp levels in plasma samples were estimated by liquid chromatography mass spectrometry (LC-MS). Cyclic Pro-Hyp levels significantly increased in the plasma after ingestion of collagen hydrolysate, reaching a maximum level after 2 h and then decreasing. The maximum level of cyclic Pro-Hyp in plasma ranged from 0.1413 to 0.3443 nmol/mL, representing approximately 5% of linear Pro-Hyp in plasma after ingestion of collagen hydrolysate. Addition of cyclic Pro-Hyp in medium at 7 nmol/mL significantly enhanced the growth rate of mouse skin fibroblasts on collagen gel more extensively compared to linear Pro-Hyp.

## 1. Introduction

Collagen is the most abundant protein in the animal body and accounts for approximately 30% of all human proteins. This protein is present as a major extracellular matrix component in the skin, muscle, cartilage, bone, and tendons. The collagen molecule forms a triple helical structure and contains a unique amino acid, hydroxyproline (Hyp), which specifically exists in collagen [1]. The heat-denatured form of collagen is called gelatin. Gelatin has a collapsed triple helical structure and has been used in foods, pharmaceuticals, cosmetics, and photographic film at the industrial scale. It has been suggested that ingestion of gelatin improves conditions of joints, skin, nails, and hair [2,3,4]. The enzymatically degraded product of gelatin, referred to as “collagen hydrolysate” or “collagen peptide”, has been developed in order to enhance the absorption and solubility of the molecule. Many recent studies have demonstrated that collagen hydrolysate ingestion also has beneficial effects on human joints and skin conditions [2,5,6,7,8,9]. Daily ingestion of collagen hydrolysate alleviates joint pain in athletes and patients with knee osteoarthritis [8,10]. Moreover, daily ingestion of collagen hydrolysate has also been shown to increase the moisture content of the epidermis in women during winter and to promote skin elasticity in women over the age of 50 [6,11]. A combination of daily ingestion of collagen hydrolysate and resistance training for 12 weeks increases muscle strength in sarcopenic patients [12]. These studies demonstrated that the ingestion of collagen hydrolysate can improve skin and joint condition as well as muscle condition, whereas no negative effects of collagen hydrolysate ingestion at 5–10 g/day for 12 weeks have been reported in human studies [13,14].

In 2005, Iwai et al. reported the discovery of Hyp-containing peptides, such as alanyl-hydroxyprolyl-glycine (Ala-Hyp-Gly), prolyl-hydroxyprolyl-glycine (Pro-Hyp-Gly), prolyl-hydroxyproline (Pro-Hyp), isoleucyl-hydroxyproline (Ile-Hyp), leucyl-hydroxyproline (Leu-Hyp), and phenilalanyl-hydroxyproline (Phe-Hyp) in human plasma after ingestion of collagen hydrolysate. They also found that Pro-Hyp was a major component among these food-derived Hyp-containing peptides [15]. Previous cell culture studies have reported on the bioactivities of Pro-Hyp. For example, Nakatani et al. reported that Pro-Hyp inhibited the mineralization of chondrocytes and modulates the expression of Runt-related transcription factor 1 (Runx1) and osteocalcin genes in a murine chondrocytic cell line [16]. Taga et al. reported that the Hyp-containing tripeptides Ala-Hyp-Gly and leucyl-hydroxyprolyl-glycine (Leu-Hyp-Gly) promoted the differentiation of MC3T3-E1 cells [17], and Ohara et al. reported that Pro-Hyp stimulated hyaluronic acid production in cultured synovium cells [13]. In addition, we have demonstrated that Pro-Hyp and hydroxyprolyl-glycine (Hyp-Gly) stimulated the growth of mouse skin fibroblasts on collagen gels [18,19]. These results partially support a mechanism whereby food-derived Hyp-containing peptides are bioactive in the human body and consequently have beneficial effects on human skin and joints after the ingestion of collagen hydrolysate.

Pro-containing dipeptides, such as prolyl-proline (Pro-Pro), prolyl-leucine (Pro-Leu), prolyl-isoleucine (Pro-Ile), prolyl-valine (Pro-Val), and prolyl-phenylalanine (Pro-Phe), have been demonstrated to form a cyclic structure by peptide bond, which is referred to as diketopiperazine [20]. Cyclic Pro-containing dipeptides exist in foods such as bread, beer, coffee, cacao, beer, and beer-brewing byproducts [21,22,23,24]. Taga et al. detected some of Hyp-containing cyclic dipeptides in ginger protease-degraded collagen hydrolysate under heating conditions [25]. Additionally, cyclic hydroxyprolyl-serine (Hyp-Ser) has been shown to alleviate hepatitis, and the cyclic forms of isoleucyl-proline (Ile-Pro), phenilalanyl-proline (Phe-Pro), Pro-Val, and leucyl-proline (Leu-Pro) have been found to exert antioxidant effects [26,27]. An in vitro study has reported the conversion of linear Pro-Hyp to cyclic Pro-Hyp, which has a blocked N-terminal region, by heating in alkaline conditions [28]. Cyclic Pro-Hyp might be generated in vivo during the digestion and absorption process of collagen hydrolysate, and may exert additional beneficial effects. However, to our best knowledge, there is no data on the presence of food-derived cyclic Pro-Hyp.

The objectives of the present study were to confirm the presence of cyclic Pro-Hyp in blood after ingestion of collagen hydrolysate in a pilot human study and to elucidate its biological activity.

## 2. Materials and Methods 

### 2.1. Collagen Hydrolysate

Collagen hydrolysate that was prepared from porcine (*Sus scrofa domesticus*) skin gelatin by enzymatic hydrolysis was a kind gift from Qsai (Fukuoka, Japan); this product can be obtained commercially. The preparation mainly consisted of peptides with a molecular weight of 3000 Da.

### 2.2. Chemicals

A standard mixture of amino acids (Type H), Hyp, acetonitrile (high-performance liquid chromatography (HPLC)-grade), trifluoroacetic acid (TFA), and phenyl isothiocyanate (PITC) were purchased from Wako Chemicals (Osaka, Japan). All other reagents were of analytical grade or better. Pro-Hyp and Hyp-Gly were purchased from Bachem (Bubendort, Switzerland). An AG 50W-×8, ion exchanger resin was purchased from Bio-Rad Laboratories (Hercules, CA, USA). The AccQ Tag was purchased from Waters (Milford, MA, USA), and consisted of the 6-aminoquinolyl-N-hydroxysuccinimidyl carbamate reagent (AccQ), acetonitrile, and 0.2 mM sodium borate buffer (pH 8.8). Dulbecco’s modified Eagle’s medium (DMEM) and Dulbecco’s phosphate-buffered saline were purchased from Sigma Chemicals (St. Louis, MO, USA). Gentamicin was obtained from Invitrogen (Carlsbad, CA, USA), and a BWT S-1820, fetal bovine serum (FBS) was obtained from Biowest (Nuaillé, France). Calf acid-soluble type I collagen solution (0.3%) was purchased from Nippi (Tokyo, Japan), and the Cell Counting Kit-8 was purchased from Dojin Glocal (Kumamoto, Japan). 

### 2.3. Preparation of Cyclic Pro-Hyp

**Figure 1:**
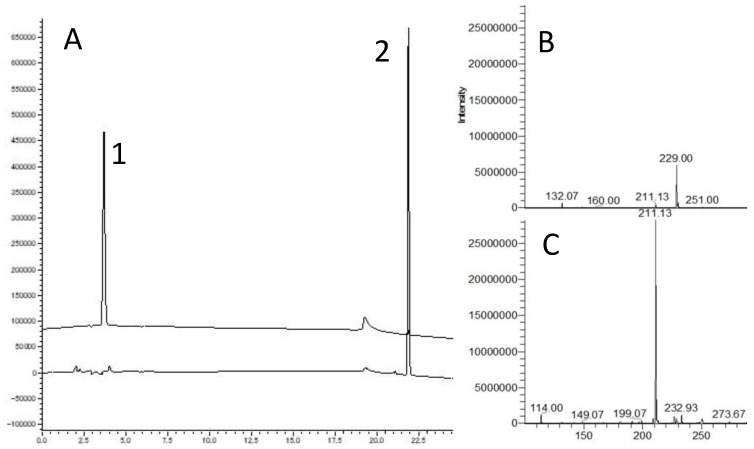
High-performance liquid chromatography (HPLC) and electrospray ionization mass spectrometry (ESI-MS) of linear and cyclic prolyl-hydroxyproline (Pro-Hyp). (**A**) The HPLC chromatogram peaks of (1) the linear Pro-Hyp and (2) after heating at 50 °C in 0.02 M ammonia solution. (**B**) Electrospray ionization mass spectra of peaks 1 and (**C**) 2 recovered from HPLC.

Cyclic Pro-Hyp was prepared according to the method of Kibrick et al. Linear Pro-Hyp was heated in 0.02 M ammonia solution at 50 °C overnight [28]. Cyclic Pro-Hyp in heated samples was isolated using an LC-20 series HPLC system (Shimadzu, Kyoto, Japan). Cyclic Pro-Hyp was resolved on an Inertsil ODS-3 250 × 4.6 mm column (GL Science, Tokyo, Japan). Binary gradient elution was performed with 0.01% TFA (solvent A) and 60% acetonitrile (solvent B) at a flow rate of 1 mL/min. The column was equilibrated with 15% B. The gradient profile was as follows: 0–30 min, 15–75% B; 30–35 min, 75–100% B; 35–40 min, 100% B; 40–40.1 min, 100–15% B; and 40.1–50 min, 15% B. The column was maintained at 45 °C, and the absorbance at 214 nm was monitored. As shown in Figure 1, Pro-Hyp, peak 1 with [M+H]^+^ ions of *m*/*z* 211.13, disappeared with the ammonia treatment and peak 2 with *m*/*z* corresponding to cyclic Pro-Hyp was generated. Cyclic peptide formation was calculated using the following equation. Cyclic formation (%) = 100 − (linear peptide peak area before heat treatment − linear peptide peak area after heat treatment)/linear peptide peak area before heat treatment × 100.

### 2.4. Human Study 

**Figure 2:**
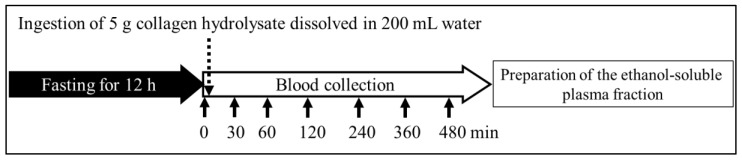
Design of the human study. Blood samples were collected before and after the ingestion of collagen hydrolysate.

The human studies were carried out according to a protocol described previously [15,29,30]. These studies were performed according to the Helsinki Declaration under the supervision of medical doctors and were approved by the experimental ethical committees of the Qsai Corporation (Fukuoka, Japan). No negative effects were reported by collagen hydrolysate ingestion at 5–10 g/day for 12 weeks in human studies [13,14] and the safety of high-dose collagen hydrolysate ingestion (1.66 g/kg body weight) was also confirmed by animal experiments [31]. The volunteers were informed of the objectives of the present study and the potential risks of ingestion of collagen hydrolysate, such as diarrhea and abdominal pain. Before the experiment, five healthy female volunteers (age 40.8 ± 9.31 years, average body weight 63.6 ± 7.33 g) fasted for 12 h and then ingested the 5 g collagen hydrolysate dissolved in 200 mL water (Figure 2). Approximately 10 mL of venous blood were collected from the cubital vein before and 30, 60, 120, 240, 360, and 480 min after the ingestion. Plasma prepared from venous blood samples was then deproteinized by adding three volumes of ethanol [13,15], and the ethanol-soluble fraction was stored at −80 °C until analysis.

### 2.5. Separation of Linear and Cyclic Pro-Hyp from Human Plasma by Strong-Cation Exchange Resin

**Figure 3:**
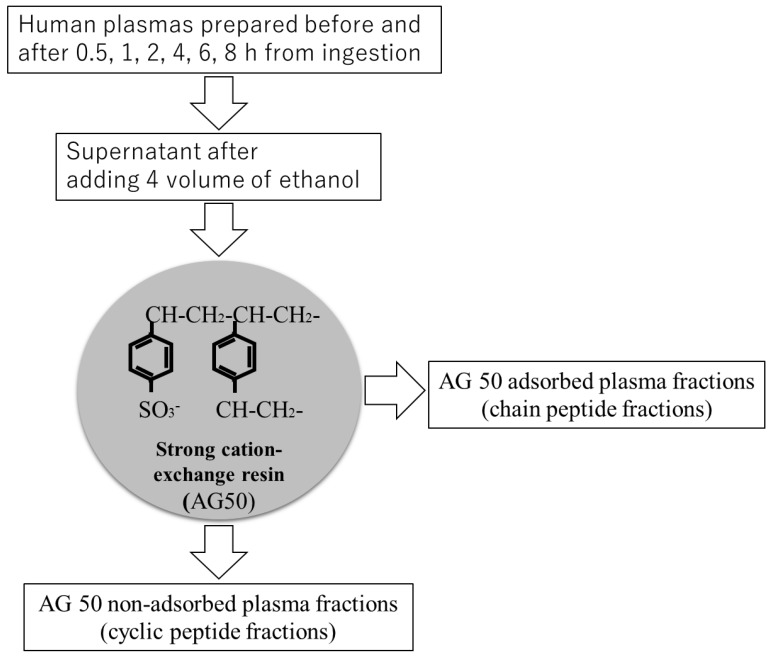
Diagram of the separation procedure for cyclic and linear peptide fractions from human plasma before and after ingestion of collagen hydrolysate.

Ethanol-soluble plasma fractions were treated with strong cation exchanger (AG50), (Figure 3). The N-terminus of the free peptides adsorbed to AG50 under acidic conditions, but those with a blocked N-terminal region did not. AG50 non-adsorbed and adsorbed plasma fractions were recovered as cyclic and linear peptide fractions, respectively. 

### 2.6. Estimation of Hyp-Containing Peptide Concentration

Amino acid analysis was performed according to the method of Bidlingmeyer et al. with slight modifications [32,33]. Amino acids in the cyclic peptide fractions of plasma were derivatized with PITC, and the resulting phenyl thiocarbamoyl amino acids were resolved on a LiChro CART 250 × 4.0 mm column (Kanto Kagaku, Tokyo, Japan) using an LC-20 series HPLC system (Shimadzu). Binary gradient elution was performed with 150 mM ammonium acetate containing 5% acetonitrile (pH 6.0; solvent A) and 60% acetonitrile (solvent B) as the mobile phases at a flow rate of 0.5 mL/min. The column was equilibrated with 100% solvent A prior to the analysis. The gradient profile was as follows: 0–0.1 min, 0% B; 0.1–1.01 min, 0–10% B; 1.01–20 min, 10–47.5% B; 20–25 min, 47.5–100% B; 25–37 min, 100% B; 37–37.1 min, 100–0% B; and 37.1–50 min, 0% B. The column was maintained at 45 °C, and the absorbance of the eluate was monitored at 254 nm. The levels of Hyp-containing peptides in the ethanol-soluble fraction were estimated by subtracting the concentration of free Hyp from that of total Hyp in the HCl hydrolysate, as described previously [13,15,19,30].

### 2.7. Liquid Chromatograph–Mass Spectrometer (LC-MS) Analysis

LC-MS was carried out using an LC-MS-2020 equipped with a Prominence HPLC system (Shimadzu) in ESI-positive ion mode. An Inertsil ODS-3 column (150 × 2.1 mm; GL Science, Tokyo, Japan) was eluted with a binary gradient of 0.01% TFA (solvent A) and 100% acetonitrile (solvent B) at a flow rate of 0.2 mL/min. The column was equilibrated with 100% A. The gradient profile was as follows: 0–2.5 min, 0% B; 2.0–15.0 min, 0–28% B; 15.0–15.1 min, 28–60% B; 15.1–20.0 min, 60%; and 20.0–30.0 min, 0% B. The column was maintained at 45 °C, and the absorbance at 214 nm was monitored. The MS was operated with nebulizer gas flow of 1.5 L/min, drying gas flow of 15 L/min, ESI voltage of 1.8 kV, temperature of 250 °C. For detection of linear and cyclic Pro-Hyp in human plasma, linear and cyclic peptide fractions were derivatized with AccQ and analyzed under the same conditions.

### 2.8. Cell Culture

BALB/c mice were purchased from SLC Japan (Shizuoka, Japan). Fibroblasts were obtained from the skin of mice as described by Rittié et al., with slight modifications [18,34]. The abdominal skin was cut into square pieces (approximately 6–7 mm in width), and eight pieces were placed at the bottom of a culture dish (75 mm in diameter) such that they were not in contact with each other. Cultivation was carried out in 8 mL DMEM containing 584 mg/L l-glutamine, 0.01 mg/mL gentamicin, and 10% FBS in a humidified incubator at 37 °C under 5% CO_2_. During cultivation, the medium was changed every 2 days. After cultivation for 2 weeks, the skin discs were removed, and fibroblasts were recovered using a 0.25% trypsin-ethylenediaminetetraacetic acid solution. The primary cultured fibroblasts were suspended to give a concentration of 5 × 10^4^ cells/mL in DMEM containing 584 mg/L l-glutamine, 0.01 mg/mL gentamicin, 10% FBS, and the test component. The fibroblasts were then incubated on a collagen gel-coated plate as described previously with 7 nmol/mL linear or cyclic Pro-Hyp. The collagen solution (0.5%) was mixed with the same volume of the double-concentrated DMEM medium containing l-glutamine, gentamicin, and the test component in the absence of FBS. The mixture (100 μL) was then poured into the wells of 96-well plastic plates, and the plates were incubated in a humidified incubator for 24 h at 37 °C under 5% CO_2_ to allow gelation. Growth of the cells on the gel after suitable intervals was estimated using Cell Counting Kit-8.

### 2.9. Statistical Analysis

The differences between the means were evaluated by analysis of variance, followed by Fisher’s protected least significant difference method (*p* < 0.05) using Excel-Toukei 2010 (Social Survey Research Information Co., Ltd. Tokyo, Japan). 

## 3. Results

### 3.1. Preparation of Cyclic Pro-Hyp

We prepared cyclic Pro-Hyp according to the method of Kibrick et al. by heating linear Pro-Hyp under alkaline conditions. As shown in Figure 1A, peaks 1 and 2 appeared in the HPLC chromatogram before and after heating of the Pro-Hyp sample, respectively. The [M+H]^+^ ions were detected at *m*/*z* 229.00 and 211.13, respectively, corresponding to the molecular weight plus H+ of linear and cyclic Pro-Hyp, respectively (Figure 1B,C). The percentage of cyclic Pro-Hyp formation compared with total Pro-Hyp before heat treatment was 97.72% ± 0.79%, and the prepared cyclic Pro-Hyp was used as a standard cyclic peptide. Additionally, we examined the percentage of cyclic Hyp-Gly formation using the same technique as for preparation of cyclic Pro-Hyp; the results showed that the rate of Hyp-Gly formation was approximately half that of Pro-Hyp (43.01% ± 1.12%).

### 3.2. Hyp Concentration in Plasma Cyclic Peptide Fractions

**Figure 4:**
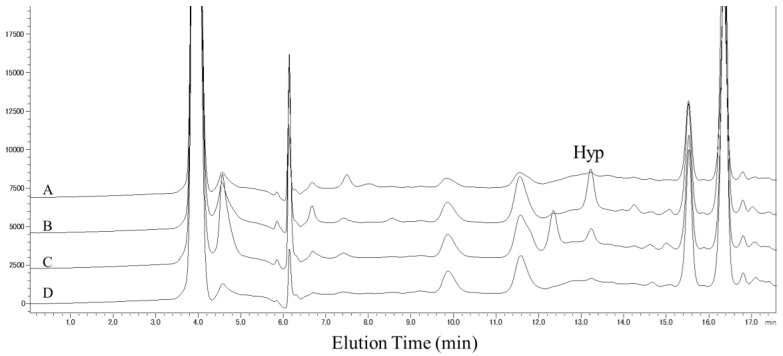
Amino acid analysis of the cyclic peptide fraction of mixed plasma samples from five volunteers. Cyclic peptide fractions prepared before (A) and at 1 (B), 2 (C), and 4 h (D) after ingestion of collagen hydrolysate were resolved by HPLC. Hydroxyproline peaks (Hyp) were observed in chromatograms of samples.

To examine the presence of cyclic Hyp-containing peptides in human plasma, Hyp-containing peptide concentrations in plasma cyclic peptide fractions of five volunteers were estimated by HPLC. As shown in the chromatogram in Figure 4, Hyp peaks in hydrolyzed cyclic peptide fractions prepared from mixed plasma of five volunteers increased at 1, 2, and 4 h after ingestion; the concentrations of cyclic Hyp peptides in these samples were 2.62, 2.27, and 1.00 nmol/mL, respectively. This result was indicative of the presence of an increase in cyclic Hyp-containing peptides in human plasma after ingestion of collagen hydrolysate. 

### 3.3. Changes in the Concentration of Cyclic Pro-Hyp in Human Plasma 

**Figure 5:**
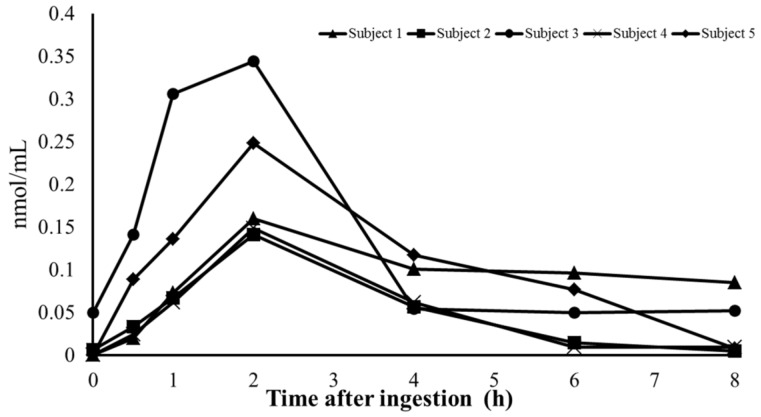
Changes in the concentration of cyclic Pro-Hyp in human plasma of five volunteers.

**Figure 6:**
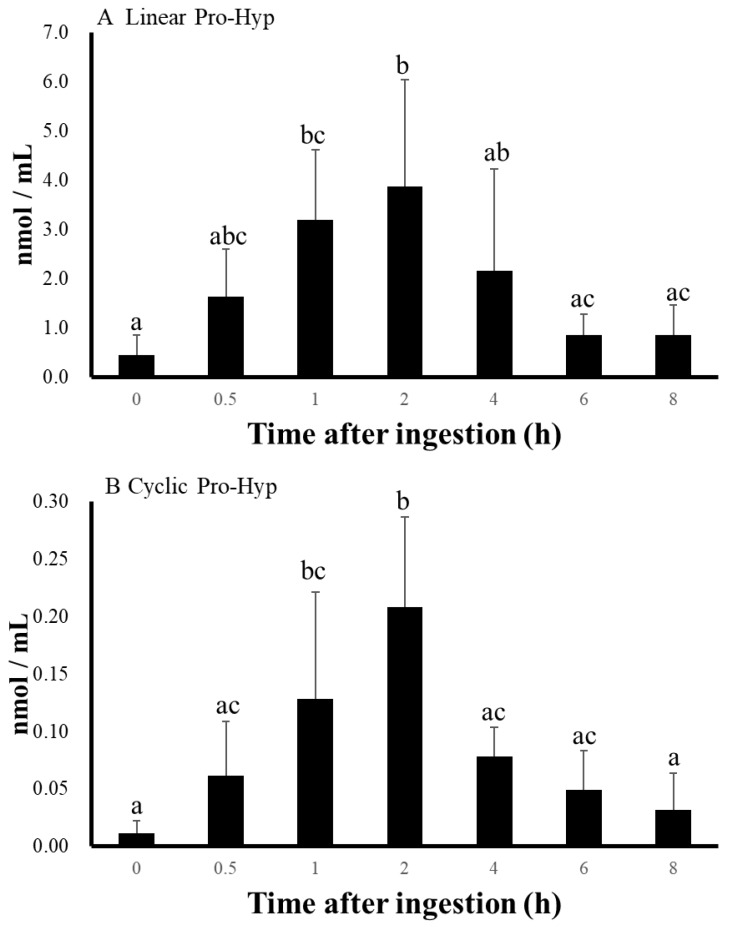
Changes in the average concentrations of linear (**A**) and cyclic (**B**) Pro-Hyp in human plasma of five volunteers. Data are shown as means ± SDs (*n* = 5). Different letters adjacent to data points indicate significant differences (*P* < 0.05).

Cyclic Pro-Hyp concentrations in the plasma of the volunteers began to increase after ingestion of collagen hydrolysate (Figure 5). The concentrations of cyclic Pro-Hyp reached a maximum after 2 h and then decreased after 4 h from ingestion of collagen hydrolysate. Differences in the maximum level of cyclic Pro-Hyp in the plasma among volunteers (0.1413–0.3443 nmol/mL) were observed, and the highest level was twice that of the lowest level, although the changes in concentrations within volunteers showed similar profiles. As shown in Figure 6, we estimated the average concentrations of linear and cyclic Pro-Hyp in human plasma after ingestion of collagen hydrolysate. The maximum levels of linear and cyclic Pro-Hyp were 3.867 and 0.2086 nmol/mL, respectively. The maximum concentration of cyclic Pro-Hyp was approximately 5% that of linear Pro-Hyp in human plasma. Changes in the concentrations of both peptides showed similar profiles, as significant increases were observed at 1 and 2 h after ingestion of collagen hydrolysate compared with that before ingestion. 

### 3.4. Cell Culture

**Figure 7:**
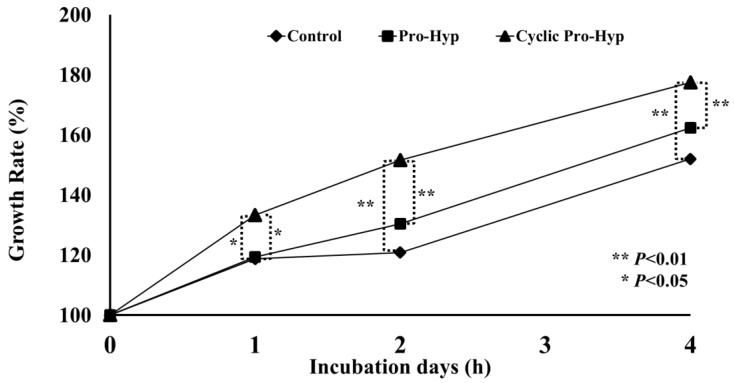
Growth rates of mouse skin fibroblasts on collagen gel. Growth rates were estimated after 0, 1, 2, and 4 days of incubation in the presence of linear (**■**) and cyclic (**▲**) Pro-Hyp or in the absence (**●**) of both peptides. ** and * indicate significant differences (*P* < 0.01 and *P* < 0.05, respectively).

To examine the bio-activities of cyclic Pro-Hyp, we examined its effect on the growth of mouse skin fibroblasts on collagen gel (Figure 7). The growth rate of skin fibroblasts on the collagen gel after incubation for 1, 2, and 4 days was significantly enhanced in the presence of cyclic Pro-Hyp compared with that observed in control cells or in fibroblasts in the presence of Pro-Hyp.

## 4. Discussion

The present study is the first report describing the detection of cyclic Pro-Hyp from human plasma after ingestion of collagen hydrolysate. Taga et al. reported the presence of cyclic Ala-Hyp and cyclic Leu-Hyp in collagen hydrolysate prepared by enzymatic digestion of ginger protease and found that ingestion of collagen hydrolysate increased the levels of cyclic Hyp-containing peptides in mouse blood [25]. In contrast, the collagen hydrolysates used in the present study contained only 0.0104% cyclic Hyp-containing peptides. This result suggested that cyclic Pro-Hyp formation may occur after digestion of collagen hydrolysate or absorption in human blood. If cyclic Pro-Hyp formation occurs in human blood, the time at which the maximum level of cyclic Pro-Hyp was reached could be delayed compared with that of linear Pro-Hyp. Thus, changes in the concentrations of both types of Pro-Hyp peptides showed similar profiles, and cyclic Pro-Hyp formation may have occurred before absorption in human blood. However, it is also possible that cyclic Pro-Hyp formation could have occurred artificially during the experimental procedures, e.g., through heat drying in ethanol. Accordingly, we estimated the concentrations of cyclic Pro-Hyp before and after heating of linear Pro-Hyp in ethanol. The contents of cyclic Pro-Hyp in total Pro-Hyp before and after heating in ethanol were approximately 0.5582% and 0.6083%, respectively. 

Because enhancement of cyclic Pro-Hyp formation during the experimental procedure was very low, cyclic Pro-Hyp, which was found in human blood in the present study, may not be an artificial cyclic peptide. Kwak et al. reported that lactic acid bacteria in foods can produce cyclic dipeptides [35,36]. Additionally, Liu et al. reported that cyclic Hyp-Ser JBP485 was absorbed by the intestine after oral administration by the intestinal peptide transporter 1 (PEPT1) [26,37]. In addition to these reports, the present results indicated that the digestion of collagen hydrolysate in digestive organs could produce cyclic Hyp-peptides and that the peptide could be absorbed via peptide transporter through the small intestine. In contrast, Taga et al. reported that cyclic Hyp-containing dipeptides could be produced by heating of linear Hyp-containing tripeptides [25]. This report also suggested the possibility for production of cyclic Hyp-containing dipeptides from the Hyp-containing tripeptides derived from digested collagen hydrolysate. 

Notably, the concentration of cyclic Hyp-containing peptides in human plasma was higher than that of cyclic Pro-Hyp, and this result indicated that other cyclic Hyp-containing peptides may exist in the human blood after ingestion of collagen hydrolysate. Over 15 Hyp-containing linear peptides have been identified in human blood after ingestion of collagen hydrolysate [38]. Thus, the cyclic forms of these Hyp-containing peptides could be increased in human blood after ingestion of collagen hydrolysate. The present results showed that cyclic Hyp-Gly formation was significantly lower than total Pro-Hyp. Different rates of cyclic peptide formation between Pro-Hyp and Hyp-Gly may cause increases in the levels of cyclic Hyp peptides in human blood after ingestion of collagen hydrolysate. Although the detailed mechanisms mediating the formation of cyclic peptides after ingestion of collagen hydrolysate have not been clarified, previous studies have reported the occurrence of cyclic dipeptides in fermented food or/and enzyme-treated products; thus, enzymatic activities may be related to cyclic dipeptide formation [35,36,39]. Therefore, differences in the maximum levels of cyclic Pro-Hyp in the blood of individual volunteers could have occurred via different efficacies of digestive enzymes for the formation of cyclic Hyp-containing peptides or via absorption of these peptides by a peptide transporter.

Our previous study demonstrated that addition of 200 nmol/mL linear Pro-Hyp in cell culture medium significantly enhanced mouse skin fibroblasts on collagen gel compared with that in the absence of Pro-Hyp [18]. The present study revealed that 7 nmol/mL cyclic Pro-Hyp caused a significant increase in the growth rates of skin fibroblasts on collagen gel compared with the same concentration of linear Pro-Hyp. This result suggested that the increase in cyclic Pro-Hyp in human blood after ingestion of collagen hydrolysate could effectively enhance wound healing process in damaged skin tissues. Differences in both types of Pro-Hyp were observed with regard to its hydrophobicity, as shown by the different elution times for HPLC separation and in the different molecular structures of the molecules. Similarly, previous reports have shown that short peptides with high hydrophobicity exhibit high uptake by cells [40], and cyclic peptide formation could enhance cell incorporation of Pro-Hyp. Although the mechanisms mediating the enhancement of fibroblast growth have not been fully clarified, differences in hydrophobicity and incorporation of both types of Pro-Hyp could be related to growth enhancement of skin fibroblasts on collagen gel. However, the concentration of cyclic Pro-Hyp detected in human blood was significantly lower than that of linear Pro-Hyp after ingestion of collagen hydrolysate. Thus, in order to enhance the efficacy of collagen hydrolysate, it is necessary to promote the absorption of cyclic Pro-Hyp in human blood. Fermentation or treatment of food-derived enzymes could enhance the occurrence and absorption of cyclic Hyp-containing peptides in human blood. Additional studies on the formation of cyclic dipeptides using food-derived enzymes and the examination of its absorption in human blood are now in progress.

## 5. Conclusions

In summary, cyclic Pro-Hyp was detected in human plasma from five volunteers after ingestion of collagen hydrolysate. The maximum level was reached 2 h after ingestion of collagen hydrolysate, and the average concentration was 0.2086 nmol/mL. Cyclic Pro-Hyp enhanced the growth rate of mouse skin fibroblasts on collagen gel. These results suggested that the increase in cyclic Pro-Hyp in human blood after ingestion of collagen hydrolysate could effectively enhance the wound healing process in damaged skin tissues.
